# The Spatial Distribution and Bioaccumulation of Anatoxin-A in Hulun Lake

**DOI:** 10.3390/toxics13110996

**Published:** 2025-11-20

**Authors:** Shiyu Li, Rui Liu, Shuhao Guo, Xiaoxuan Chen, Wenxue Wu, Bo Pang, Zixuan Liu, Haiming Ying, Yanlong Zhang, Yuanyuan Zhang, Chengxue Ma

**Affiliations:** 1 College of Wildlife and Protected Area, Northeast Forestry University, Harbin 150040, Chinazhangynlng68@hotmail.com (Y.Z.); 2 Heilongjiang Key Laboratory of Complex Traits and Protein Machines in Organisms, Harbin 150040, China; 3 Hulunbuir Academy of Inland Lakes in Northern Cold & Arid Areas, Hulunbuir 165456, China

**Keywords:** Hulun lake, cyanobacterial bloom, anatoxin-a, spatial distribution

## Abstract

The intensification of eutrophication in global water bodies has exacerbated the occurrence of cyanobacterial blooms, whose secondary metabolites can have detrimental effects on animals, humans, and ecosystems. This study analyzed and statistically evaluated the species composition and distribution of phytoplankton, assessed the concentration of anatoxin-a (ATX-a) in environmental and biological samples (n = 261), and explored the spatial distribution and bioaccumulation of ATX-a in Hulun Lake (Inner Mongolia, China). In late July 2024, the cyanobacteria *Dolichospermum* spp. comprised 85.5% of the total phytoplankton density. ATX-a levels were vertically distributed with higher concentrations in deeper water (3 m; 146.69 ± 11.84 ng·L^−1^) and sediments (3.28 ± 0.45 ng·g^−1^ dry weight) as compared to surface layers (0.5 m; 132.46 ± 8.19 ng·L^−1^). In fish, bioaccumulation of ATX-a was greatest in the liver (2.37 ± 1.85 ng·g^−1^), followed by intestinal contents (1.83 ± 0.74 ng·g^−1^), with minimal accumulation in muscle tissues (1.74 ± 0.77 ng·g^−1^). ATX-a levels were higher in smaller fish (minnows) than larger fish (Predatory carp, Gibel carp, and European carp). Additionally, all fish tissue samples contained ATX-a, suggesting that aquatic organisms were continuously exposed to ATX-a throughout the summer. A biodilution of ATX-a was observed from phytoplankton (384.82 ± 176.82 ng·L^−1^) to zooplankton (1.27 ± 0.12 ng·g^−1^), followed by biomagnification from zooplankton to fish.

## 1. Introduction

In recent years, frequent outbreaks of harmful algal blooms and eutrophication of water bodies have been observed due to human activities [1,2]. Global warming exacerbates eutrophication and intensifies cyanobacterial blooms by creating ideal conditions—specifically warmer waters, stronger stratification, and greater nutrient inputs—that promote their proliferation. Moreover, cyanobacterial blooms have tended to occur earlier and in water bodies at higher latitudes [3]. As a result, toxic cyanobacterial blooms, which are rarely observed in high-latitude lakes, have become more frequent in recent years and are often composed of multiple species [3]. Compared to lower-latitude lakes with higher water temperatures, cold-water lakes at higher latitudes exhibit elevated concentrations of the neurotoxin Anatoxin-a (ATX-a), with a statistically significant correlation between toxin levels and latitude. Specifically, northern Lithuanian lakes demonstrated the highest ATX-a concentrations and very high biomass of *Dolichospermum* spp. and *Aphanizomenon gracile*. ATX-a was consistently absent in warm lakes but present in colder counterparts. Alpine lakes showed analogous trends of increasing ATX-a prevalence [4], suggesting cold-water ecosystems are more vulnerable to neurotoxin accumulation than warmer systems [5]. As compared to lakes in southern China, outbreaks of cyanobacterial blooms in high-latitude lakes in northern China have received much less attention. Since 1986, Hulun Lake has experienced large-scale and prolonged cyanobacterial blooms [6,7], drawing increasing attention to this issue. Among the dominant species in Hulun Lake, *Dolichospermum* spp. and other toxic cyanobacteria are capable of producing harmful metabolic byproducts, such as ATX-a and geosmin [8], which are toxic and detrimental to both the environment and human health.

ATX-a, a potent, fast-acting neurotoxin first isolated from *Anabaena flos-aquae* [9], acts as a powerful cholinergic depolarizing neuromuscular blocker on the postsynaptic membrane. Symptoms of ATX-a poisoning in animals include muscle fasciculations, opisthotonus, respiratory muscle spasms, and salivation [10,11]. Ingestion of ATX-a leads to gastrointestinal and neurological disorders, liver, kidney, and muscle damage [12] and even increased risks of various cancers [13,14]. Numerous studies have confirmed that ATX-a is also toxic to aquatic organisms [8]. Bioaccumulation of ATX-a in fish can eventually manifest as neurotoxicity, immunotoxicity, and cytotoxicity [15]. For instance, the guppy (*Poecilia reticulata*) will suffocate upon exposure to ATX-a at 20 μg·g^−1^ (oral absolute lethal dose) within 1–2 min due to respiratory paralysis [16]. The oral median lethal dose of ATX-a in juvenile carp is reportedly 11.50 μg·g^−1^ [16]. ATX-a can also cause acute toxicity in humans. In 2002, a case of poisoning and death due to ATX-a was reported in Wisconsin, USA, which involved a young victim who swam in a pond experiencing an algal bloom, ingested contaminated water, and later exhibited neurological symptoms. ATX-a and *Anabaena flos-aquae* were detected in the stomach contents of the deceased [16]. Given the hazardous nature of this toxin, many countries have begun monitoring ATX-a levels in water [17,18,19].

The persistence of ATX-a in aquatic environments is closely related to the duration of the cyanobacterial species producing ATX-a [20]. After cyanobacteria produce ATX-a, it typically remains temporarily inside the cells. Once the cyanobacteria cells age and lyse, a large amount of ATX-a is released into the water. However, under specific conditions (such as low-light environments), ATX-a may leak from unlysed cells [21]. The release of ATX-a toxins into the water is influenced by various environmental factors, such as pH, light exposure, and sediment adsorption. Under acidic conditions with pH < 3, ATX-a is relatively stable and can be preserved for longer periods [22]. In water with pH 8–9, ATX-a degrades within 1–2 h under sunlight, losing its toxicity [23]. In addition, sediment adsorption is an important natural pathway for the fate of ATX-a. Once adsorbed by sediments, ATX-a may be rapidly degraded by bacteria and protozoa inhabiting the sediments [21]. Reports indicate that the half-life of ATX-a in sediments is approximately 5–10 days [24]. However, these sedimented toxins may still be re-released into the upper water column before they are fully degraded [21].

Although reference values for acute short-term exposure to ATX-a in drinking water and recreational water are 30 and 60 μg·L^−1^, respectively, the World Health Organization acknowledges that the current toxicological database for ATX-a is insufficient to support the issuance of formal guideline values [25]. However, systematic monitoring of ATX-a in China remains sparse, resulting in poorly quantified contamination levels in Hulun Lake. Crucially, scientific literature lacks documentation of ATX-a occurrence, spatiotemporal distribution, and trophic transfer dynamics in arid endorheic lakes, particularly within representative hydrosystems such as Hulun Lake. This deficiency constitutes a critical knowledge gap for temperate Asia’s dryland aquatic ecosystems. Therefore, the aim of the present study was to assess the extent of ATX-a contamination in Hulun Lake to provide a scientific basis to develop algal toxin early warning and monitoring strategies.

## 2. Materials and Methods

### 2.1. Study Area

Hulun Lake, the fifth largest lake in China and the largest in northern China [7], is located in the western part of the Hulunbuir Grassland, with geographic coordinates ranging from 116°58′ to 117°47′ East longitude and 48°40′ to 49°20′ North latitude [7]. The total area of the lake is approximately 2339 km^2^, with an average depth of 5.7 m and a maximum depth of 8 m [26]. The lake is typically ice-covered for 170–180 days during the winter. The annual average temperature is relatively low, and the water is alkaline. From 2014 to 2022, cyanobacterial blooms in Hulun Lake generally occurred between late June and late August, with the majority observed in July and August [27].

**Figure 1:**
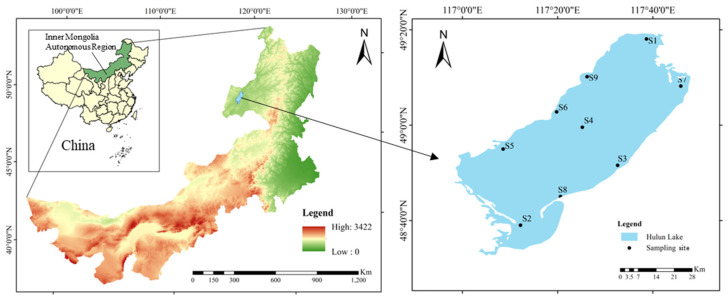
Study area and sampling sites of Hulun Lake.

The locations of the environmental and biological samples collected in late July 2024 in this study are shown in Figure 1. Sampling sites were established using a grid-based uniform design to ensure spatial representativeness, encompassing nine designated sites (S1–S9). Sampling site coordinates are provided in Table S1. The northeastern region of Hulun Lake is designated as the Golden Coast Scenic Tourist Area (S1). The lake contains three fishing zones (S6, S7, and S8, with S7 representing the core area of the lake). Surrounding the lake are extensive natural pastures, which support various livestock, including camels, sheep, horses, and cattle [7]. The lake and related tributary rivers serve as primary sources of drinking water. Water, sediment, phytoplankton, zooplankton, and fish samples were collected from nine sites (S1–S9) in the region. 261 samples were obtained. Each sampling site consists of 3 water samples, 1 sediment sample, 1 zooplankton sample, and 24 fish samples comprising 12 minnows (*Hemiculter bleekeri*), 6 Predatory carp (*Cultrichthys erythropterus*), 3 Gibel carp (*Carassius auratus gibelio*), and 3 European carp (*Cyprinus carpio*).

Sampling was conducted at each sampling site at the end of July, a time when the frequency of algal blooms was relatively high. The study provides a snapshot of the season, focusing solely on ATX-a.

### 2.2. Density and Identification of Phytoplankton

Water and phytoplankton samples were collected at a water surface depth of 0.5 m in 500 mL brown glass bottles. Phytoplankton samples (500 mL) were fixed in 15 mL of 5% Lugol’s iodine solution. After 48 h of sedimentation in Utermöhl chambers, the supernatant was concentrated to 50 mL, and cell numbers were counted using an upright optical microscope (ICC50 W; Leica Microsystems, Wetzlar, Germany) at 400× magnification (10 × eyepiece; 40 × objective lens) [28]. Cell quantification of the concentrated samples (1 mL) was conducted with a Sedgewick-Rafter counting chamber. Cell abundances (cells·L^−1^) were determined from at least triplicate counts per sample [29]. In this study, the taxonomy of phytoplankton followed the classification described in the relevant literature [30].

### 2.3. Sample Collection and Preparation

At each site, 20 L of water was collected and filtered through a 25-μm plankton net to concentrate the phytoplankton. The resulting concentrated suspension was made up to a final volume of 50 mL with filtered lake water and stored in a 50 mL amber glass vial [31]. In the laboratory, an aliquot of this concentrate was further processed to remove large particles like zooplankton. Specifically, a subsample was filtered onto pre-combusted and pre-weighed GF/C filters (1.2-μm pore size) for subsequent analysis [32].

Water samples (500 mL) were collected from depths of 0.5 and 3 m in Lake Hulun, stored in brown glass bottles, and filtered through 0.45-µm water-grade fiber filter membranes (NAVIGATOR, Lab Instrument Co., Ltd., Tianjin, China) [33].

Sediment core samples (2 kg) were collected from each sampling site using a sediment sampler, placed in light-protective, self-sealing bags. Sediment samples were transported to the laboratory at 4 ± 1 °C. The sediment was removed from the freezer and spread evenly on a glass plate, then air-dried in the dark at room temperature for 48 h before analysis [32].

At each site, zooplankton were concurrently collected by filtering 20 L of water through a separate 25-μm plankton net. The entire concentrated zooplankton sample was transferred to a 50 mL specimen bottle and preserved [31]. In the laboratory, the sample was centrifuged at 3000 rpm for 15 min to concentrate the zooplankton biomass prior to toxin analysis. In the laboratory, the samples were centrifuged at 3000 rpm for 15 min to separate the zooplankton from the water. To avoid contamination or overestimation of ATX-a concentrations due to the presence of phytoplankton, the collected zooplankton was filtered and washed repeatedly with distilled water using a 25 µm mesh and observed under a stereoscopic microscope (ICC50 W; Leica Microsystems) to ensure that the sample was free of cyanobacteria [34].

At each sampling site (S1–S9), 12 minnows, 6 predatory carp, 3 Gibel carp, and 3 European carp were captured using bottom nets, surface nets, and fish traps. The fish were maintained at 4 ± 1 °C until transportation to the laboratory, where the size and weight of each specimen was recorded. Due to substantial disparities in fish body size, samples were prepared by pooling multiple individuals to meet the minimum biomass requirement for analytical procedures. Specifically, four Minnows were pooled to constitute one sample, two Predatory carp were combined into one sample, while Gibel carp and European carp were processed individually as one sample per fish, with three replicates prepared for each sample. The fish were sampled on ice and ATX-a was extracted from the livers, muscle tissues, and intestinal contents.

### 2.4. Chemicals and Standards

The ATX-a water standard solution used was purchased from Cifga Laboratories S.A. (Lugo, Spain) [35]. The ATX-a standard solution with a concentration of 5.1 μg·mL^−1^ was diluted into a gradient using 0.1% acetic acid in water. According to the supplier’s instructions, both the commercial stock solution and the prepared stock solution were stored in a freezer at −20 °C.

### 2.5. Extraction of ATX-A from Samples

Following the freeze-drying of 1 g (wet weight) aliquots of each biological sample—including phytoplankton, zooplankton, fish liver, intestinal contents, and muscle tissue—the resulting dry material was pulverized into a fine powder—was individually pulverized into a fine powder using a tissue lyser with 5 mm stainless steel beads (Qiagen., Valencia, CA, USA). Subsequently, 10 mL of methanol containing 0.01% acetic acid was added to each powdered sample, followed by vortex mixing for 1 min. The mixtures were then centrifuged at 4 °C and 12,000× *g* for 10 min. The resulting supernatants were collected and concentrated to dryness under a gentle stream of N_2_ (30 °C). The residues were reconstituted in 1 mL of methanol and stored at 4 °C pending further analysis [36,37,38].

A 100 mL aliquot of the water sample was first filtered through a 0.45-µm hydrophilic fiber filter (NAVIGATOR, Lab Instrument Co., Ltd.) to remove algae cells, suspended particles, and other impurities. Subsequently, formic acid was added to the filtered sample as a stabilizer at a final concentration of 0.1% (*v*/*v*) to prevent degradation of the target analyte, followed by thorough mixing before further use. Water samples were extracted using 150 mg cation-exchange solid-phase extraction cartridges (Oasis MCX; Waters Corporation, Milford, MA, USA). Each MCX cartridge was conditioned with 5 mL of methanol and equilibrated with 5 mL of reagent-grade water. After sample loading, the cartridge was dried under vacuum for at least 10 min. Retained analytes were eluted with 6 mL of a 5% (*v*/*v*) ammonium hydroxide solution in methanol. The eluate was evaporated to dryness under a stream of nitrogen at 30 °C using a RapidVap-N_2_ system (Labconco, Kansas City, MO, USA) with rotation set at 80 rpm for 60 min. The resulting residue was reconstituted in 1 mL of reagent-grade water prior to analysis [36].

Sediment (1 g dry weight equivalent) were accurately weighed into 15 mL centrifuge tubes. Each sample underwent two consecutive extraction cycles, with each cycle consisting of the following steps: addition of 4 mL of methanol containing 200 mM ammonium acetate, vigorous vortex mixing for 0.5 min at 3500 rpm, ultrasonication for 15 min, and centrifugation at 6000 rpm for 10 min. The resulting supernatants from both cycles were combined and gently concentrated to a volume of less than 0.2 mL under a stream of nitrogen at 30 °C. The concentrated extract was then reconstituted with 2 mL of HPLC-grade water, followed by vortex mixing and filtration through GHP membrane filters using microsyringe filter holders. The filtered extract was further concentrated and subsequently analyzed by online solid-phase extraction coupled with liquid chromatography–tandem mass spectrometry (LC-MS/MS) [39,40].

### 2.6. ATX-A Detection and Quantification

The chromatographic analysis was performed on the SCIEX Exion LC (Framingham, MA, USA) liquid chromatography system. An ACQUITY UPLC HSD T3 column (2.1 × 100 mm, particle size 1.8-μm, Waters, Manchester, UK) was used. During the analysis, the column was maintained at 35 °C, and the injection volume was 100-μL. Mobile phase A was an acetonitrile solution, and mobile phase B was a Milli-Q solution containing 0.1% formic acid (FA). Gradient elution was carried out according to the conditions outlined in the Table S2 [35].

Quantification of cyanotoxins was performed using a Triple Quad 5500+ triple quadrupole mass spectrometer (LC-MS/MS) in dynamic multiple reaction monitoring (MRM) mode. The mass spectrometer was operated in positive electrospray ionization (ESI) mode, and the detection ion voltages were set according to the conditions outlined in the Table S3. The determination of analyte recovery rates across different sample matrices is presented in the Table S4.

The method demonstrated excellent linearity (R^2^ > 0.996) across all matrices, with limits of detection (LOD) and quantification (LOQ) determined as follows: water (LOD = 20 ng·L^−1^, LOQ = 60 ng·L^−1^), biological tissues (LOD = 472 ng·L^−1^, LOQ = 1430 ng·L^−1^), and sediment (LOD = 332 ng·L^−1^, LOQ = 1005 ng·L^−1^).

### 2.7. The Total Length and Weight of the Fish

The total length and weight of minnows (n = 108), Predatory carp (n = 54), Gibel carp (n = 27), and European carp (n = 27) were measured using a caliper and a balance, respectively. The scientific names, feeding habits, and habitat information of the fish species are provided in Table 1. Information on the diet and habitat strata of the four fish species is available in the relevant literature [41,42].

### 2.8. Calculation of Biological Magnification Effects

To assess whether organisms can bioaccumulate ATX-a from their diets, a biomagnification factor (BMF) was adopted to evaluate the biomagnification potential of these compounds along the food web [43,44]. Original concentrations were used to calculate BMF. Studies employing dissolved toxins or aqueous cyanobacterial extracts were not included in the analysis [45,46]. Detailed equations are given below:

$$BMF=C_{predator}/C_{prey}$$

where *C_predator_* (ng·g^−1^ ww) and *C_prey_* (ng·g^−1^ ww) are the ATX-a concentration in predator and prey, respectively.

### 2.9. Risk Assessment

The human health risks of ATX in lake water was assessed based on hazard quotients (HQs) and the risk quotients (RQs) was used for ecological risk assessment of the ATX in lake water in this study. Detailed methods and calculation formulas are described in Section S9 of the Supplementary Materials.

### 2.10. Statistical Analysis

Spatial analysis utilized ArcMap 10.8, employing kriging interpolation to generate phytoplankton and ATX-a distribution maps. Data visualization and graphical representations were created using Origin 2024. Statistical analyses were performed in IBM SPSS Statistics 27. Pearson correlation analysis assessed relationships between variables, with significance defined as *p* < 0.05 or *p* < 0.01. Group comparisons employed one-way ANOVA (with LSD post hoc test) and independent samples t-tests, considering *p* < 0.05 or *p* < 0.01 significant [47]. Data are presented as mean ± standard deviation (SD). The assumptions of normality and homoscedasticity for all parametric tests (ANOVA and Pearson correlation) were verified prior to analysis. Detailed results of these verification tests are provided in Tables S6 and S7.

## 3. Results

### 3.1. Species Abundances

**Figure 2:**
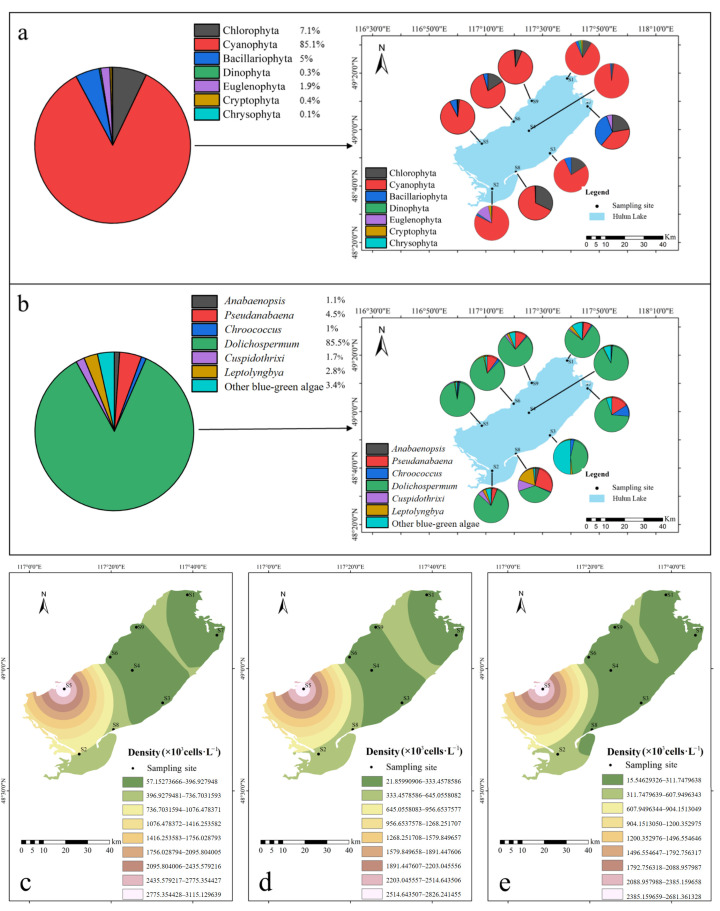
Phytoplankton composition and spatial distribution in Hulun Lake (0.5 m). (**a**) Phylum composition of phytoplankton in Hulun Lake and the phylum distribution at each sampling site. (**b**) Composition of Cyanophyta in Hulun Lake and the distribution at each sampling site. (**c**) Spatial Distribution Characteristics of Phytoplankton. (**d**) Spatial Distribution Characteristics of Cyanophyta. (**e**) Spatial Distribution Characteristics of *Dolichospermum* spp.

In total, 7 phyla, 59 genera of phytoplankton were identified. Specifically, the Chlorophyta (green algae) comprised 23 genera, Cyanophyta (blue-green algae) included 12 genera, Bacillariophyta (diatoms) consisted of 15 genera, Dinophyta (dinoflagellates) included 1 genus, Euglenophyta (euglenoids) contained 5 genera, Cryptophyta (cryptomonads) consisted of 1 genus, and Chrysophyta (golden algae) comprised 2 genera. Cyanophyta was the dominant phylum, accounting for 85.1% of the total phytoplankton population (Figure 2a). The most dominant species was *Dolichospermum* spp. from the Nostocales order of Cyanophyta, which accounted for 85.5% of all cyanobacteria (Figure 2b). The common cyanobacterial species in Hulun Lake are shown in Figure S1.

During the study period, Cyanophyta, Chlorophyta, and Bacillariophyta dominated the phytoplankton community (Figure 2a). Sampling site S2 exhibited the highest diversity, encompassing seven phyla: Chlorophyta, Cyanophyta, Bacillariophyta, Euglenophyta, Dinophyta, Cryptophyta, and Chrysophyta. Site S5 had the highest phytoplankton density, reaching 2.85 × 10^6^ cells·L^−1^ (Figure 2c). In terms of cell density, Cyanophyta dominated, with an average density of 5.09 × 10^5^ cells·L^−1^, followed by Chlorophyta and Bacillariophyta, with cell densities of 4.25 × 10^4^ and 2.99 × 10^4^ cells·L^−1^, respectively. Cyanobacterial bloom intensity was significantly higher in the southern lake region compared to the north, with the most severe outbreaks occurring in the southwest (Figure 2c–e).

### 3.2. Detection of ATX-A Concentration in Cyanobacterial Cells

**Figure 3:**
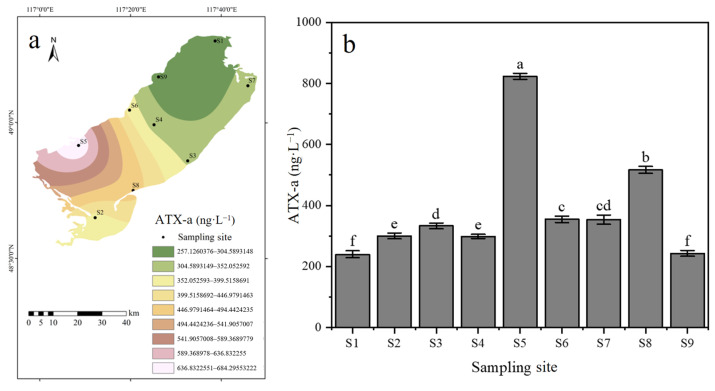
Spatial Distribution and Heterogeneity Analysis of Intracellular ATX-a in Cyanobacterial Cells. (**a**) The spatial distribution of intracellular ATX-a. (**b**) Spatial Heterogeneity of ATX-a Concentrations (n = 3). Note: Data labeled with different letters indicate a significant difference (*p* < 0.05).

The average concentration of ATX-a in cyanobacterial cells from Hulun Lake was 384.82 ± 176.82 ng·L^−1^, with the highest concentration (823.55 ± 15.2 ng·L^−1^) detected at sampling site S5 and the lowest (239.93 ± 7.19 ng·L^−1^) at site S1. (Figure 3a,b). The concentrations of intracellular ATX-a at sampling site S5 was significantly higher than those at other sampling sites (*p* < 0.05).

### 3.3. Spatial Distribution of ATX-A

**Figure 4:**
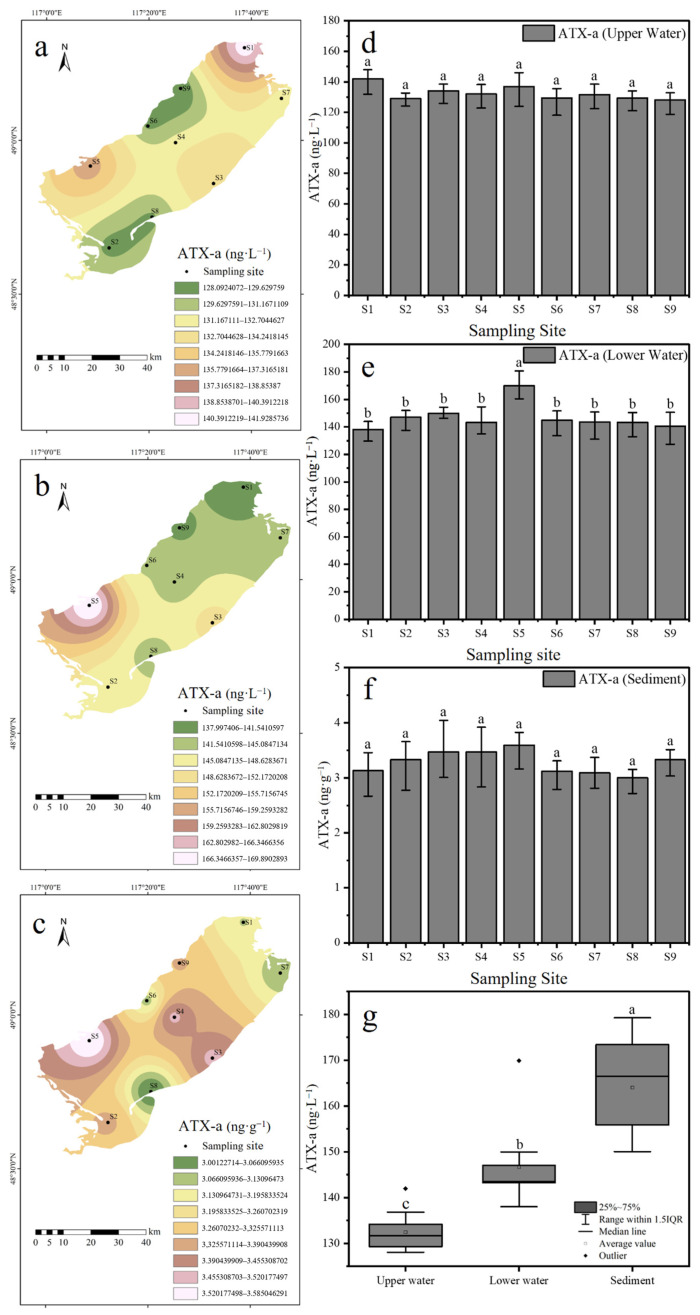
Spatial distribution of ATX-a. (**a**) Spatial distribution of ATX-a (0.5 m). (**b**) Spatial distribution of ATX-a (3 m). (**c**) Spatial distribution of ATX-a (Sediment). (**d**) ATX-a concentration in surface water (0.5 m; n = 3). (**e**) ATX-a concentration in deep water (3 m; n = 3). (**f**) ATX-a concentration in sediment (n = 3). (**g**) The concentration of ATX-a at different depths of the water column (n = 27). Note: In figure (**g**), the sediment units were converted from ng·g^−1^ to ng·L^−1^ to facilitate comparison with water sample data. Data labeled with different letters indicate a significant difference (*p* < 0.05).

In late July 2024, the concentration of ATX-a equivalents in the upper water layers of Hulun Lake ranged from 129.00 ± 4.46 to 136.81 ± 11.47 ng·L^−1^, with an average concentration of 132.46 ± 8.19 ng·L^−1^. In the lower water layers, ATX-a concentrations ranged from 138.00 ± 7.45 to 169.90 ± 8.34 ng·L^−1^, with an average concentration of 146.69 ± 11.84) ng·L^−1^. The concentration of ATX-a in the sediments varied from 3.00 ± 0.25 to 3.59 ± 0.46 ng·g^−1^, with an average of 3.28 ± 0.45 ng·g^−1^. At the surface layer of the lake (0.5 m), the highest concentration of ATX-a was observed at sampling site S1. In the deeper water (3 m) and sediment, the highest concentrations of these secondary metabolites were observed at sampling site S5 (Figure 4a–c). In the lower water column, the ATX-a concentration at sampling site S5 exhibited a significant difference as compared to other sampling sites (*p* < 0.05). However, no significant differences were observed between the sampling sites in the upper water column and sediment. (*p* > 0.05; Figure 4d–f).

### 3.4. Measurement of Fish Total Length and Weight

The total length and weight of four fish species were measured. Minnows had the smallest total length and weight among the four species, measuring 92.09 ± 45.91 mm and 8.46 ± 3.05 g, respectively, followed by the Predatory carp (total length, 179.80 ± 56.9 mm; weight, 55.22 ± 46.82 g), Gibel carp (total length, 204 ± 40.44 mm; weight, 126.86 ± 73.76 g), and European carp (total length, 237 ± 41.38 mm; weight, 186.29 ± 96.6 g; Figure S2). There were significant differences in the total length and weight among the four fish species (*p* < 0.05).

### 3.5. Bioaccumulation of ATX-A

**Figure 5:**
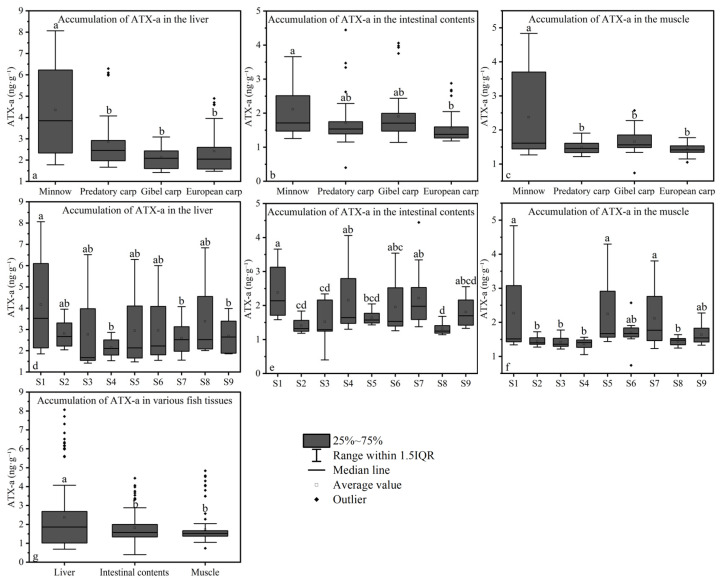
Accumulation of ATX-a in fish samples. (**a**–**c**). Accumulation of ATX-a in the liver, intestinal contents, and muscle, respectively (n = 27). (**d**–**f**). Accumulation of ATX-a in the liver, intestinal contents, and muscle, respectively (n = 12). (**g**) Accumulation of ATX-a in various fish tissues (n = 108) Note: Data labeled with different letters indicate a significant difference (*p* < 0.05).

ATX-a was quantified in all animal samples (n = 135). In fish tissues, ATX-a accumulated in liver, muscle, and intestinal contents. Hepatic accumulation was highest in minnows (4.35 ± 2.12 ng∙g^−1^), followed by predatory carp (2.86 ± 1.32 ng∙g^−1^), European carp (2.42 ± 1.08 ng∙g^−1^), and gibel carp (2.12 ± 0.51 ng∙g^−1^; Figure 5a). In intestinal contents, minnows exhibited the highest mean ATX-a concentration (2.11 ± 0.81 ng∙g^−1^), followed by gibel carp (1.90 ± 0.80 ng∙g^−1^), predatory carp (1.71 ± 0.75 ng∙g^−1^), and European carp (1.57 ± 0.46 ng∙g^−1^; Figure 5b). Muscle tissue accumulation was also greatest in minnows (2.37 ± 1.29 ng∙g^−1^), with lower levels in gibel carp (1.66 ± 0.35 ng∙g^−1^), predatory carp (1.48 ± 0.19 ng∙g^−1^), and European carp (1.43 ± 0.17 ng∙g^−1^; Figure 5c). The mean ATX-a concentration in zooplankton was 1.27 ± 0.12 ng∙g^−1^.

**Figure 6:**
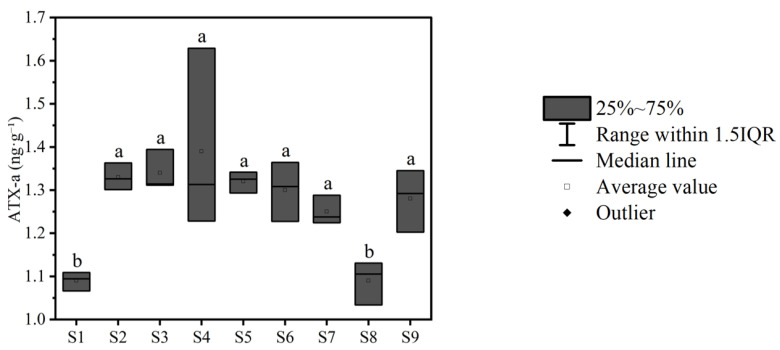
The accumulation of ATX-a in zooplankton at each sampling site (n = 3). Note: Data labeled with different letters indicate a significant difference (*p* < 0.05).

Significant interspecific differences in hepatic ATX-a bioaccumulation were observed between minnows and the other three fish species (*p* < 0.05; Figure 5a). In intestinal contents, ATX-a levels differed significantly between minnows and European carp (*p* < 0.05; Figure 5b). Muscle tissue ATX-a accumulation also differed significantly between minnows and the other three species (*p* < 0.05; Figure 5c). Spatially, hepatic ATX-a accumulation at site S1 differed significantly from sites S4, S7, and S9 (*p* < 0.05; Figure 5d). Intestinal content ATX-a levels at S1 differed significantly from sites S2, S3, and S8 (*p* < 0.05; Figure 5e). Significant differences in muscle ATX-a accumulation were found between sites S1, S5, S7 and sites S2, S3, S4, S8 (*p* < 0.05; Figure 5f). Overall, ATX-a accumulation followed the hierarchy: liver > intestinal contents > muscle tissue. Significant differences in accumulation were found between liver and both intestinal contents and muscle tissues (*p* < 0.05; Figure 5g). In contrast, zooplankton ATX-a accumulation showed no significant spatial variation across sites (*p* > 0.05; Figure 6).

### 3.6. Subsection Biomagnification of ATX-A

Biomagnification patterns of ATX-a exhibited significant trophic-level dependence in Hulun Lake’s food web (Table S9). Zooplankton demonstrated biodilution relative to intracellular cyanotoxins (BMF =0.0054 ± 0.0028) In contrast, all piscine consumers displayed pronounced hepatic biomagnification when feeding on zooplankton prey. Minnow liver tissue showed the highest biomagnification factor (BMF = 4.770 ± 0.391). Predatory carp followed with a BMF of 2.033 ± 0.147, while European carp and gibel carp exhibited BMFs of 1.764 ± 0.093 and 2.591 ± 0.244. The consistent BMF values exceeding unity in fish consumers (range: 1.764 ± 0.093–4.770 ± 0.391) confirm significant trophic amplification from zooplankton to predatory species.

### 3.7. Correlations of Environmental Concentrations and Biological Accumulation of ATX-A

**Figure 7:**
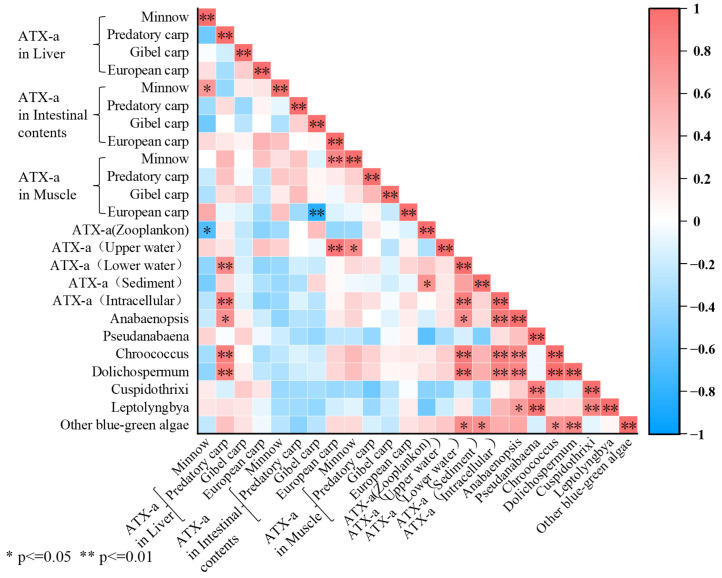
Pearson Correlation Analysis. Correlation was considered statistically significant at *p* < 0.05 and *p* < 0.01.

Pearson correlation analysis was conducted to examine the relationship between phytoplankton density, environmental ATX-a concentrations, and ATX-a bioaccumulation (Figure 7). Minnow hepatic ATX-a showed strong correlations with surface water (0.5 m) ATX-a and intracellular ATX-a (both, *p* < 0.01). Lower water (3 m) ATX-a correlated strongly with intracellular ATX-a and *Dolichospermum* spp. density (both, *p* < 0.01). Additionally, *Chroococcus* density correlated significantly with both lower water ATX-a and intracellular ATX-a (both, *p* < 0.01).

### 3.8. Risk Assessment Reveals Low Human Health and Ecological Risks

The ecological and human health risks associated with ATX-a concentrations in Lake Hulun were quantitatively assessed using the Hazard Quotient (HQ) and Risk Quotient (RQ) methodology. Detailed methods and calculation results are provided in Table S8 of the Supplementary Materials.

The measured environmental concentration (MEC) of ATX-a across all nine sampling sites (S1–S9) exhibited limited spatial variability, ranging from 0.126 ± 0.009 μg·L^−1^ to 0.146 ± 0.002 μg·L^−1^ (Table S8). Site S7 recorded the lowest mean concentration, while Site S3 showed the highest.

For human health risk assessment, all calculated Hazard Quotient (HQ) values were substantially below the safety threshold of 1.0. The HQ for adults ranged from 0.013 to 0.015, while that for children ranged from 0.004 to 0.005, indicating negligible risk through direct water exposure for both demographic groups.

Regarding ecological risk, the Risk Quotient (RQ) values for the sensitive aquatic organisms remained well below the critical level of 1.0. The RQ for Zooplankton (PNEC = 5.0 μg·L^−1^) varied between 0.025 and 0.029, and for Cyprinus carpio (PNEC = 8.33 μg·L^−1^), it ranged from 0.015 to 0.018. All values were classified as “Low” risk, suggesting a minimal probability of adverse effects on the assessed aquatic species under current contamination levels.

## 4. Discussion

This study aimed to investigate the spatial distribution characteristics of summer cyanobacterial blooms and ATX-a, as well as the bioaccumulation of ATX-a in water and biological samples from different locations in Hulun Lake. Unlike previous studies, this research revealed the distribution patterns of ATX-a in the specific ecosystem of Hulun Lake, emphasizing the key species responsible for harmful algal blooms, rather than treating cyanobacterial blooms as a homogeneous entity. Additionally, evidence of potential ATX-a bioaccumulation and transfer along the food chain was found.

In-depth investigations across diverse regions have examined cyanotoxin distribution and biomagnification. Studies in the Zemborzycki Reservoir (eastern Poland) reported a general positive correlation between cyanotoxin concentrations and cyanobacterial bloom density [31]. Cyanotoxin persistence (e.g., ATX-a, saxitoxins) in aquatic environments depends partially on the persistence of cyanobacterial species [20], aligning with the present findings. Furthermore, studies in Washington state (USA) freshwater lakes demonstrated tissue-specific cyanotoxin accumulation (liver > intestine > muscle) [48], consistent with the pattern observed in Hulun Lake. However, similar to microcystin transfer in other lakes [49,50].

The summer cyanobacterial blooms in Hulun Lake are primarily concentrated in the southwestern corner (S5), expanding outward and leading to high cyanobacterial density in the south. The southwestern sector of Hulun Lake exhibits a semi-enclosed bay morphology, characterized by severely restricted water exchange capacity with flow velocities typically below 0.01 m·s^−1^. Under such hydrodynamic conditions, filamentous cyanobacteria leverage gas vesicles for buoyancy regulation, facilitating vertical aggregation in quiescent waters. This mechanism drives high-density cyanobacterial accumulation in the southwestern region [51].

ATX-a, an endotoxin released upon cell membrane rupture [52], can also be actively released during exponential growth in response to environmental factors like low light [21,53,54]. Analysis revealed a significant correlation (*p* < 0.01) between intracellular ATX-a concentration and the abundance of *Dolichospermum* spp. and *Chroococcus* (Figure 6). Conversely, ATX-a distribution in surface waters was irregular, showing no significant correlation (*p* > 0.05) with levels in deeper water/sediments or specific cyanobacterial abundance (Figure 6). This irregularity is attributed to accelerated ATX-a photodegradation in the high-light upper layer (0.5 m; Secchi depth: 39.67 ± 0.58 to 45 ± 1 cm; Table S5) compared to deeper water (3 m) [22,55]. Light flux is significantly higher in the upper water layer (0.5 m) than the deeper water layer (3 m), resulting in photodegradation of ATX-a in surface waters and an irregular distribution pattern. In contrast, ATX-a concentrations in deeper water (3 m) were significantly correlated with the abundance of *Dolichospermum* spp. and *Chroococcus* (both, *p* < 0.01; Figure 6). However, due to the significantly lower density of *Chroococcus* compared to *Dolichospermum* spp., *Dolichospermum* spp. is considered the primary potential producer of ATX-a in Hulun Lake. Nevertheless, *Chroococcus* and other cyanobacteria remain important potential producers of ATX-a that should not be overlooked. Vertically, the concentration of ATX-a is lower in surface water (0.5 m) than deeper water (3 m; Figure 4g). At deeper water depths, the effect of light flux on ATX-a degradation is diminished, and the toxin was adsorbed by suspended particles in the water, settling into the sediment [56]. The highest concentration of ATX-a was detected in the lake sediment, followed by the deeper water layers, while the surface water contained the lowest concentration of ATX-a, showing a trend of increasing ATX-a concentrations with depth. The maximum total ATX-a concentration detected in the water column of Hulun Lake was 193.9 ng·L^−1^ We referenced the Italian National Guidelines for Cyanobacterial Blooms, which classify a water body as being in an “Emergency Phase” when the concentration of ATX-a exceeds 20 ng·L^−1^ (20,000 ng·L^−1^). From a direct human health perspective for recreational or drinking water use (after standard treatment), the situation in Hulun Lake is not at a critical emergency level [57].

During the trophic transfer of ATX-a through food chains, zooplankton often act as intermediaries and during algal blooms are forced to consume toxic cyanobacteria, thereby encapsulating the cyanotoxins, which are subsequently transferred to higher trophic levels [58]. However, in Hulun Lake, the dominant species of rotifers and cladocerans accounted for over 69% of the total zooplankton density [31]. The average size of rotifers and cladocerans is less than 1 mm, and these species are not well-suited to feed on cyanobacteria [59]. As compared to fish species at higher trophic levels, zooplankton at lower trophic levels are significantly less affected by cyanobacterial toxins in terms of inhibited growth, reduced grazing, failure to thrive, and bioaccumulation [15]. As a result, lower levels of ATX-a were detected in the zooplankton samples, suggesting a lower impact on zooplankton than fish.

While fish bioaccumulation typically follows a feeding strategy hierarchy (zooplanktivores < carnivores < herbivorous planktivores < omnivores; [49], this pattern was absent in Hulun Lake. Significant interspecific differences in fish length and weight (*p* < 0.05; Figure S2), coupled with reduced biotransformation/excretion efficiency in smaller fish [60], suggest ATX-a accumulation is size-dependent. Supporting this, minnows exhibited higher hepatic, intestinal, and muscular ATX-a accumulation than the three larger species. The amount of ATX-a in minnow muscles also correlated strongly with the concentration of ATX-a in surface waters (*p* < 0.05; Figure 6), aligning with their feeding ecology (Table 1). Interestingly, despite both being omnivorous, European carp and Gibel carp displayed distinct accumulation profiles: European carp showed the lowest ATX-a in muscle and intestinal contents, while Gibel carp exhibited the lowest hepatic accumulation, indicating superior metabolic capacity. Gibel carp exhibits higher CYP450 substrate affinity than European carp, with cytochrome P450 enzymes (especially CYP3A4) efficiently catalyzing ATX-a hydroxylation. This converts ATX-a into low-toxicity metabolites and accelerates their elimination. Conversely, European carp shows lower hepatic detoxification enzyme activity, prolonging toxin retention and resulting in higher hepatic ATX-a residues [61]. In parallel, Gibel carp’s elongated intestinal architecture reduces toxin transit velocity, prolonging luminal residence time and consequently enhancing ATX-a retention in intestinal digesta relative to European carp. Predatory carp (carnivorous) differed significantly in size from the omnivorous species (*p* < 0.05) but showed no significant differences in tissue ATX-a accumulation (*p* > 0.05; Figure 5a–c). Across all species, ATX-a levels consistently followed: liver > intestinal contents > muscle, further evidencing potential trophic transfer.

Results confirm persistent ATX-a in the Hulun Lake environment and its continuous bioaccumulation within aquatic organisms. Although alkaline conditions facilitate natural ATX-a degradation [23], sustained concentrations occur during blooms, particularly at depths >3 m. Co-occurring microcystins synergistically enhance ATX-a toxicity [20,62], elevating risks for humans, livestock, and wildlife consuming contaminated water [63]. While medaka (*Oryzias latipes*) studies show complete hepatic ATX-a elimination within one metabolic cycle (10 days) [16], 100% of fish sampled from Hulun Lake over 15 days tested positive for ATX-a in liver, intestinal contents, and muscle. This indicates chronic, long-term ATX-a exposure. Muscle tissue accumulation further confirms trophic transfer. In the food chain of Hulun Lake, the dominant zooplankton in Hulun Lake are rotifers and cladocerans, which are typically less than 1 mm in size [31]. In contrast, the filamentous cyanobacterium *Dolichospermum* spp. can form large colonies that significantly exceed the ingestible size range for these small zooplankters. This creates a physical feeding barrier, drastically limiting the direct consumption and transfer of intracellular ATX-a from the toxin-producing phytoplankton to the zooplankton. Despite its water solubility, ATX-a can accumulate in specific fish tissues. As a potent neurotoxin, it binds with high affinity to nicotinic acetylcholine receptors. The liver, as a primary site for detoxification and blood filtration, becomes a major reservoir. The continuous dietary intake from non-selective feeding, coupled with the high-affinity, target-site binding that reduces elimination from key organs, leads to an uptake rate that can exceed the depuration rate [64]. This results in a net accumulation and the observed organ-specific biomagnification (BMF > 1) in fish liver. ATX-a transfer from cyanobacteria to zooplankton is characterized by biodilution, while subsequent transfer to fish demonstrates biomagnification.

This study also evaluated the potential risks of ATX-a to humans and aquatic organisms in Lake Hulun, with all calculated Hazard Quotient (HQ) values being substantially below 0.1. Water samples collected from various depths at all sampling sites indicated a low health risk to both adults and children. Furthermore, similar to the human health risks, ATX-a posed low ecological risks to both carp and zooplankton at all sampling sites, with all Risk Quotient (RQ) values far below 0.1 (Table S8).

Chronic low-concentration ATX-a exposure still poses health risks through fish consumption. Additionally, cyanobacterial ingestion reduces fish nutritional quality and flavor [63,64], impairing local fisheries. Consequently, cyanotoxin co-occurrence in Hulun Lake aquatic fauna demands urgent attention.

## 5. Conclusions

In late July 2024, persistent detection of harmful cyanobacterial species occurred in Hulun Lake, with severe events in the southern region dominated by the filamentous *Dolichospermum* spp. Results identify *Dolichospermum* spp. and *Chroococcus* as the primary potential ATX-a producers. Despite alkaline lake water conditions during summer months, persistent toxic blooms maintain a continuous ATX-a presence in water and sediments. ATX-a distribution at 0.5 m depth showed no significant spatial variation but increased with depth. ATX-a transfers from cyanobacterial cells through the food chain, accumulating in zooplankton and fish. This bioaccumulation presents health risks to livestock, wildlife, and humans, with particular concern about elevated dietary exposure from high intake of small fish species such as minnows. These findings establish a critical baseline for ATX-a dynamics in arid inland lakes, offering methodological frameworks to assess cyanotoxin risks in climatically vulnerable freshwater ecosystems under global warming scenarios.

## Figures and Tables

**Table 1:** Feeding habits and habitat depths of four fish species in Hulun Lake.

English Name	Scientific Name	Diet	Habitat Strata
Minnow	*Hemiculter bleekeri*	Planktivore	Upper
Predatory carp	*Cultrichthys erythropterus*	Piscivore	Middle
Gibel carp	*Carassius auratus gibelio*	Omnivore	Upper
European carp	*Cyprinus carpio*	Omnivore	Bottom

## Data Availability

The data presented in this study are available from the corresponding author upon reasonable request.

## Supplementary Materials

The following supporting information can be downloaded at https://www.mdpi.com/article/10.3390/toxics13110996/s1: Table S1. Geographic coordinates of sampling sites S1–S9. Table S2. Mobile phase and elution gradient. Table S3. Mass spectrometer ion voltage conditions setting. Table S4. Recovery and RSD of ATX-a in different matrices. Figure S1. Common cyanobacterial in Hulun Lake. Figure S2. The total length and weight of the fish. Table S5. Transparency parameters and pH of the Hulun Lake aquatic environment. Table S6. Shapiro–Wilk test. Table S7. Levene test. Table S8: Site-Specific Human Health and Ecological Risk Assessment for ATX-a in Hulun Lake. Table S9. Summary of BMF for ATX-a (Wet Weight Basis).
