# Supplementary material for: The Spatial Distribution and Bioaccumulation of Anatoxin-A in Hulun Lake
Source: Toxics. 2025 Nov 20;13(11):996. doi: 10.3390/toxics13110996 (PMC12656580; doi:10.3390/toxics13110996)
Supplement: Supplementary file 1 [file toxics-13-00996-s001.zip › toxics-3936636-supplementary.pdf]

Supplementary Material

The spatial distribution and bioaccumulation of Anatoxin-a in Hulun Lake

Shiyu Li [1, 2], Rui Liu [1, 2], Shuhao Guo [1, 2], Xiaoxuan Chen [1, 2], Wenxue Wu[1, 2],

Bo Pang [3], Zixuan Liu [3], Haiming Ying [1], Yanlong Zhang [1], Yuanyuan Zhang [1, 2 #],

Chengxue Ma [1 #]

1. College of Wildlife and Protected Area, Northeast Forestry University, Harbin 150040, PR China

2. Heilongjiang Key Laboratory of Complex Traits and Protein Machines in Organisms, Harbin 150040, PR China

3. Hulunbuir Academy of Inland Lakes in Northern Cold & Arid Areas, Hulunbuir 165456, PR China

[#] Corresponding Authors.

College of Wildlife and Protected Area, Northeast Forestry University, Harbin, China.

Email: Yuanyuan Zhang: yuanyuan85945@126.com

Chengxue Ma: mch007@vip.163.com

Contents:

1. Geographic locations of sampling sites in Hulun Lake
2. Mobile phase and elution gradient
3. Mass spectrometer ion voltage conditions setting
4. Calculation of sample recovery
5. The common cyanobacterial in Hulun Lake
6. The total length and weight of the fish
7. Transparency parameters and pH of the Hulun Lake aquatic environment
8. Shapiro–Wilk test and Levene test
9. Risk assessment
10. Summary of BMF for ATX-a

1. Geographic locations of sampling sites in Hulun Lake

Table S1. Geographic coordinates of sampling sites S1-S9

Sampling site	Longitude (E)	Latitude (N)
S1	117.644490	49.301510
S2	117.764700	49.136650
S3	117.543610	48.859780
S4	117.343390	48.750890
S5	117.203490	48.650510
S6	117.142570	48.916680
S7	117.329880	49.046750
S8	117.436520	49.169290
S9	117.419850	48.992790

2. Mobile phase and elution gradient

Table S2. Mobile phase and elution gradient

**Time （min）**	**Flow （mL/min）**	**A （%） Acetonitrile**	**B （%） 0.1% formic acid (FA)**
0.50	0.3	80	20
1.50	0.3	80	20
2.50	0.3	80	20
2.60	0.3	80	20
3.50	0.3	80	20

3. Mass spectrometer ion voltage conditions setting

Table S3. Mass spectrometer ion voltage conditions setting

Substance	Parent Ion/Fragment Ion	Collision-Voltage (V)	Collision Energy (V)
ATX-a	166.167/149.2	18	18

4. Method Validation: Linearity, Limits of Detection and Quantification, Accuracy, and Precision

（1）Linearity, LOD, and LOQ

The linearity, limit of detection (LOD), and limit of quantification (LOQ) of the method were initially assessed using matrix-matched standard calibrations. Separate standard curves were established for water, sediment, and biological samples (including fish tissues, phytoplankton, and zooplankton). The LOD and LOQ were calculated based on the standard curve method using the formulas LOD = 3.3 × S_a / b and LOQ = 10 × S_a / b, where S_a is the standard deviation of the y-intercept and b is the slope of the regression line. The method demonstrated excellent linearity (R^2 > 0.996) across all matrices. The method demonstrated varying sensitivity across matrices, with limits of detection (LOD) and quantification (LOQ) determined as follows: water (LOD = 19.9 ng/L, LOQ = 60.2 ng/L), biological tissues (LOD = 472 ng/L, LOQ = 1430 ng/L), and sediment (LOD = 332 ng/L, LOQ = 1005 ng/L).

（2）Accuracy and Precision

Sample preparation was performed according to the matrix-specific procedures outlined in Sections 2.4–2.6. The deuterated internal standard ATX-a-d5 was added to all samples prior to extraction to correct for procedural losses and matrix effects. Specifically, 1 mL of ATX-a-d5 at a concentration of 100 ng/L was added to each water sample (n = 3), while 50 µL of the same internal standard solution (100 ng/L) was added to sediment and biological tissue samples (n = 3). Water samples were then extracted

using Oasis MCX cartridges preconditioned with methanol and reagent-grade water. Biological tissues were processed by mechanical disruption with a tissue lyser, centrifugation, and nitrogen-assisted concentration. Sediment samples underwent sequential extraction using methanolic ammonium acetate, followed by filtration and concentration.

Analysis was carried out on a SCIEX Exion LC system fitted with an ACQUITY UPLC HSD T3 column (2.1 × 100 mm, 1.8 μm) maintained at 35 °C. The injection volume was 100 μL, and gradient elution was performed using 0.1% formic acid in water (mobile phase B) and acetonitrile (mobile phase A), as specified in Table 1. Quantification was based on external calibration with internal standard correction.

Recovery was determined by comparing measured ATX-a concentrations to the theoretical spiked values, and precision was expressed as the relative standard deviation (RSD) of triplicate measurements. Method blanks and solvent controls were analyzed concurrently to monitor contamination. All recoveries fell within 70–120% with RSDs below 15%, confirming the method's reliability for quantifying ATX-a in various environmental and biological matrices at realistic concentrations.

Table S4. Recovery and RSD of ATX-a in different matrices

Samples	Mean Recovery（%）	RSD（%）
Liver	78.02 ± 0.65	0.83
Intestinal contents	98.73 ± 6.83	6.91
Muscle	103.83 ± 6.50	6.22
Zooplankon	78.48 ± 1.20	0.31
Sediment	98.73 ± 0.87	0.42
phytoplankton	93.61 ± 0.73	0.66
Upper water	87.13 ± 0.95	0.84

5. The common cyanobacterial in Hulun Lake

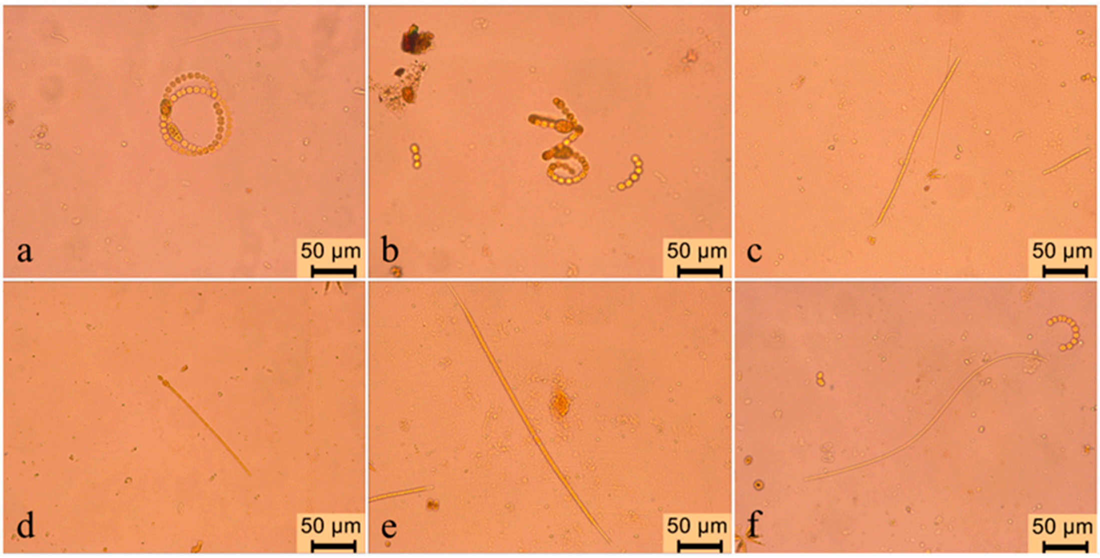

Fig. S1. Common cyanobacterial in Hulun Lake. (a) *Dolichospermum.* (b) *Dolichospermum.* (c) *Pseudanabaena*. (d) *Cylindrospermopsis*. (e) *Cuspidothrix* i*ssatschenkoi*. (f) *Leptolyngbya.*

6. The total length and weight of the fish

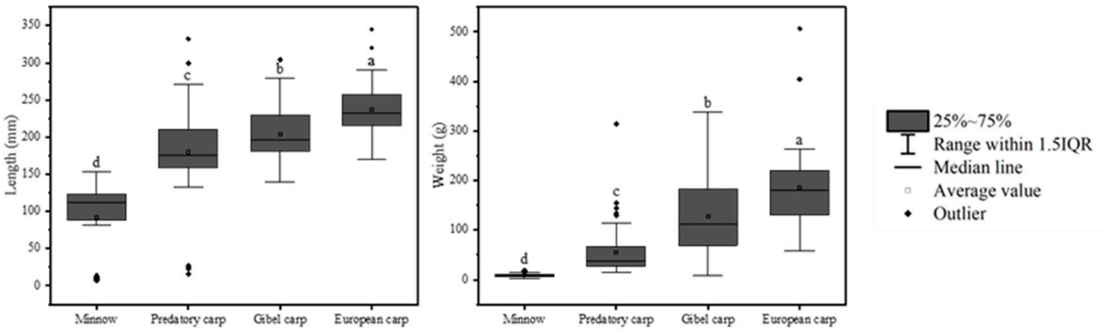

Fig. S2. The total length and weight of the fish. Minnow (n=108). Predatory carp (n=54). Gibel carp (n=27). European carp (n=27). Note: Data labeled with different letters indicate a significant difference (p < 0.05).

7. Transparency parameters and pH of the Hulun Lake aquatic environment

The pH value was measured using a Pen-type pH meter PH-100 (Shanghai Lichen Bangxi Instrument Technology Co., Ltd.)., with a sampling depth of 0.5 meters. Transparency (SD) was measured using a Secchi disk. Each sampling site was measured three times for repetition.

Table S5. Transparency parameters and pH of the Hulun Lake aquatic environment

Sampling site	SD (cm)	pH
S1	39.67±0.58	8.68±0.021
S2	37	8.59±0.067
S3	45±1	8.66±0.015
S4	38.67±0.58	8.69±0.01
S5	36.5±0.5	8.72±0.006
S6	42.5±0.5	8.62±0.01
S7	41	8.37±0.021
S8	37.67±0.58	8.74±0.031
S9	41±1	8.77±0.015

8. Shapiro–Wilk test and Levene test

Table S6. Shapiro–Wilk test

Variable Name	Data transformation	Shapiro–Wilk p Value
ATX-a in the liver (Minnows)	Logarithmic transformation	0.2135
ATX-a in the liver (Predatory carp)	-	0.3955
ATX-a in the liver (Gibel carp)	-	0.8186
ATX-a in the liver (European carp)	Logarithmic transformation	0.1872
ATX-a in the intestinal contents (Minnows)	-	0.3886
ATX-a in the intestinal contents (Predatory carp)	Logarithmic transformation	0.0894
ATX-a in the intestinal contents (Gibel carp)	Logarithmic transformation	0.1567
ATX-a in the intestinal contents (European carp)	-	0.5027
ATX-a in the muscle (Minnows)	-	0.0717
ATX-a in the muscle (Predatory carp)	-	0.8006
ATX-a in the muscle (Gibel carp)	-	0.1241
ATX-a in the muscle (European carp)	-	0.9203
ATX-a (Zooplankon)	-	0.7669
ATX-a (Intracellular)	-	0.0952
ATX-a (Upper Water)	-	0.95
ATX-a(Lower Water)	-	0.9054
ATX-a (Sediment)	-	0.4529
Anabaenopsis	Box-Cox transformation (λ=0.35)	0.1274
Pseudanabaena	Box-Cox transformation (λ=0.42)	0.1562
Chroococcus	Logarithmic transformation	0.1238
Dolichospermum spp.	Logarithmic transformation	0.0673
Cuspidothrixi	Box-Cox transformation (λ=0.28)	0.0987
Leptolyngbya	Logarithmic transformation	0.0942
Other blue-green algae	Logarithmic transformation	0.1789

Table S7. Levene test

Variable Name	Number of Groups	Levene Statistic	P Value
ATX-a in the liver (Minnows)	9	2.145	0.087
ATX-a in the liver (Predatory carp)	9	1.876	0.134
ATX-a in the liver (Gibel carp)	9	1.234	0.342
ATX-a in the liver (European carp)	9	1.543	0.215
ATX-a in the intestinal contents (Minnows)	9	1.987	0.112
ATX-a in the intestinal contents (Predatory carp)	9	2.456	0.056
ATX-a in the intestinal contents (Gibel carp)	9	1.678	0.173
ATX-a in the intestinal contents (European carp)	9	1.324	0.291
ATX-a in the muscle (Minnows)	9	1.543	0.215
ATX-a in the muscle (Predatory carp)	9	1.128	0.391
ATX-a in the muscle (Gibel carp)	9	1.432	0.247
ATX-a in the muscle (European carp)	9	1.659	0.173
ATX-a (Zooplankon)	9	1.215	0.335
ATX-a (Intracellular)	9	2.134	0.079
ATX-a (Upper Water)	9	1.325	0.291
ATX-a (Lower Water)	9	1.457	0.235
ATX-a (Sediment)	9	1.679	0.17

9. Risk assessment

The human health risks of ATX in lake water was assessed based on hazard quotients (HQs) [1, 2]. HQ was calculated from Eq.

$$HQ = CDI/RfD \quad (1)$$

assuming the human ingested the lake water :(1) where CDI (μg/kg body weight) is the chronic daily intake by ingestion of cyanotoxins per unit body weight as per Eq. RfD (μg/(kg·d)) represents the tolerable daily intake (TDI) for cyanotoxins. There is no specific guideline advised in the TDI value of ATX by the WHO. Herein, the TDI value of anatoxin-a (0.1 μg/(kg·d)) was used to evaluate the health risks of ATX, according to the Oregon Public Health Division (OPHD) guideline.

$$CDI = \frac{C*IR}{BW} \quad (2)$$

where C (μg/L) is the level of cyanotoxins, IR is the water intake rate (1 L/day for a child, 2 L/day for an adult)), and BW is the body weight (10 kg for a child, 60 kg for an adult). $HQ < 0.1$, $0.1 \leq HQ < 1$, and $HQ > 1$ represent a low risk, a moderate risk, and a high risk, respectively (Xiang et al., 2019; Wan et al., 2020).

The risk quotients (RQs) were used for ecological risk assessment of the ATX in lake water in this study [3, 4]. RQ was calculated from Eqs. (3), (4).

$$RQ = \frac{MEC}{PNEC} \quad (3)$$

$$PNEC = \frac{NOEC}{AF} \quad (4)$$

where MEC (μg/L) is the measured environment concentration of cyanotoxins; PNEC

is the predicted no-effect concentration; NOEC is the no observed-effect concentration; AF is the assessment factor. The value of AF is 10 in this study [4]. 0.01 ≤ RQ < 0.1, 0.1 ≤ RQ < 1, and RQ ≥ 1 represent a low risk, a moderate risk, and a high risk, respectively.

Table S8: Site-Specific Human Health and Ecological Risk Assessment for ATX-a in Hulun Lake

**Sampling Site**	**MEC (μg/L) (Mean ± SD)**	**HQ (Adult)**	**HQ (Child)**	**RQ (Zooplankton)** (PNEC = 5.0 μg/L)	**RQ (Cyprinus carpio)** (PNEC = 8.33 μg/L)
S1	0.131 ± 0.004	0.013 (Low)	0.004 (Low)	0.026 (Low)	0.016 (Low)
S2	0.144 ± 0.003	0.014 (Low)	0.005 (Low)	0.029 (Low)	0.017 (Low)
S3	0.146 ± 0.002	0.015 (Low)	0.005 (Low)	0.029 (Low)	0.018 (Low)
S4	0.131 ± 0.010	0.013 (Low)	0.004 (Low)	0.026 (Low)	0.016 (Low)
S5	0.141 ± 0.011	0.014 (Low)	0.005 (Low)	0.028 (Low)	0.017 (Low)
S6	0.142 ± 0.010	0.014 (Low)	0.005 (Low)	0.028 (Low)	0.017 (Low)
S7	0.126 ± 0.009	0.013 (Low)	0.004 (Low)	0.025 (Low)	0.015 (Low)
S8	0.139 ± 0.008	0.014 (Low)	0.005 (Low)	0.028 (Low)	0.017 (Low)
S9	0.141 ± 0.009	0.014 (Low)	0.005 (Low)	0.028 (Low)	0.017 (Low)

The PNEC (Predicted No-Effect Concentration) data for both zooplankton and carp were obtained from the ECOTOX database.（https://cfpub.epa.g ov/ecotox/search.cfm）

1 **References**

[1] Q. Xue, A.D. Steinman, L. Xie, L. Yao, X. Su, Q. Cao, Y. Zhao, Y. Cai, Seasonal variation and potential risk assessment of microcystins in the sediments of Lake Taihu, China, Environmental Pollution, 259 (2020) 113884.

[2] H. Li, X. Gu, H. Chen, Z. Mao, R. Shen, Q. Zeng, Y. Ge, Co-occurrence of multiple cyanotoxins and taste-and-odor compounds in the large eutrophic Lake Taihu, China: Dynamics, driving factors, and challenges for risk assessment, Environmental Pollution, 294 (2022) 118594.

[3] A. Derbalah, R. Chidya, W. Jadoon, H. Sakugawa, Temporal trends in organophosphorus pesticides use and concentrations in river water in Japan, and risk assessment, Journal of Environmental Sciences, 79 (2019) 135-152.

[4] C. Cheng, A.D. Steinman, K. Zhang, Q. Lin, Q. Xue, X. Wang, L. Xie, Risk assessment and identification of factors influencing the historical concentrations of microcystin in Lake Taihu, China, Journal of Environmental Sciences, 127 (2023) 1-14.

10. Summary of BMF for ATX-a

Table S9. Summary of BMF for ATX-a (Wet Weight Basis).

Trophic Transfer	BMF (Mean ± SD)	n	t-value	df	p-value	Statistical Conclusion
Phytoplankton→ Zooplankton	0.0054 ± 0.0028	27	-183.5	26	< 0.0001	Significant biodilution
Zooplankton →Minnow (liver)	4.770 ± 0.391	27	50.27	26	< 0.0001	Significant biomagnification
Zooplankton →Predatory carp (liver)	2.033 ± 0.147	27	36.47	26	< 0.0001	Significant biomagnification
Zooplankton →Gibel carp (liver)	1.764 ± 0.093	27	42.54	26	< 0.0001	Significant biomagnification
Zooplankton →European carp (liver)	2.591 ± 0.244	27	33.71	26	< 0.0001	Significant biomagnification
